# Multi-environment trials data analysis: linear mixed model-based approaches using spatial and factor analytic models

**DOI:** 10.3389/frma.2025.1472282

**Published:** 2025-04-11

**Authors:** Tarekegn Argaw, Berhanu Amsalu Fenta, Habtemariam Zegeye, Girum Azmach, Assefa Funga

**Affiliations:** ^1^Climate and Computational Science Research Directorate, Ethiopian Institute of Agricultural Research (EIAR), Addis Ababa, Ethiopia; ^2^College of Agriculture & Environmental Science-African Sustainable Agriculture Research Institute (ASARI), University Mohammed VI Polytheistic (UM6P), Ben Guerir, Morocco; ^3^Kulumsa Agricultural Research Center, EIAR, Assela, Ethiopia; ^4^Bako Agricultural Research Center, EIAR, Bako, Ethiopia; ^5^Debre Zeit Agricultural Research Center, EIAR, Debre Zeit, Ethiopia

**Keywords:** multi-environment trials, linear mixed models, spatial analysis, spatial + G × E analysis, genetic gain

## Abstract

The analysis of multi-environment trials (MET) data in plant breeding and agricultural research is inherently challenging, with conventional ANOVA-based methods exhibiting limitations as the complexity of MET experiments grows. This study presents linear mixed model-based approaches for MET data analysis. Ten MET grain yield datasets from national variety trials in Ethiopia were used. Randomized complete block (RCB) design analysis, spatial analysis, and spatial+genotype-by-environment (G × E) analysis were compared under linear mixed model framework. Spatial analysis detected significant local, global, and extraneous spatial variations, with positive spatial correlations. For the spatial + G × E analysis, increasing the order of the factor analytic (FA) models improved the explanation of G × E variance, though the optimal FA model order was dataset-dependent. Integrating spatial variability through the spatial + G × E modeling approach substantially improved genetic parameter estimates and minimized residual variability. This improvement was particularly notable in larger datasets, where the number of trials and the size of each trial played a crucial role for presence of spatial variability and strong GxE effects. Additionally, the genetic correlation heat maps and dendrograms provided intuitive insights into trial relationships, revealing patterns of strong positive, negative, and weak correlations, as well as distinct trial clusters. The results clearly demonstrate that linear mixed model-based approaches, especially the spatial + G × E analysis excel in capturing complex spatial plot variation and G × E effects in MET data by effectively integrating spatial and FA models. These insights have important implications for improving the efficiency and accuracy of MET data analysis, which is crucial for improving genetic gain estimation in plant breeding and agricultural research, ultimately accelerating the delivery of high-performing crop varieties to farmers and consumers.

## 1 Introduction

Crop variety development is a fundamental pillar of modern agriculture, with advancements over the past century playing a vital role in enhancing global food security, improving farmer livelihoods, and promoting sustainable farming practices (Qaim, 2020; Begna and Begna, 2021). By creating high-performing, adaptable cultivars that are resilient to biotic and abiotic stresses, researchers have enabled stable and abundant crop yields, bolstered food supplies, and supported the economic wellbeing of farming communities, while also facilitating the adoption of other sustainable agricultural innovations (Zsögön et al., 2022; Swarup et al., 2021). Continued investment and innovation in this field will be crucial as the world navigates the complex challenges of ensuring long-term food security and environmental sustainability (Blakeney, 2022).

Multi-environment trials (METs) are a crucial element of the crop variety development pipeline. In this process, newly bred crop genotypes are evaluated across a range of agro-ecological environments (Smith et al., 2001b, 2021; Brown et al., 2020). The primary purpose of METs is to capture the influence of diverse environmental factors on the expression of genotypic performance. This allows for the assessment of G × E effects- a critical consideration in identifying high-performing and stable crop varieties that can perform well under a range of conditions (Verbyla, 2023; Lee et al., 2023; Lisle et al., 2021).

Understanding G × E is a fundamental aspect of plant breeding, as it provides insights into how different genotypes respond to diverse environmental conditions. This interaction is critical because it directly affects important agronomic outcomes, such as yield and the ability of plants to adapt to varying climates and soil types (Van Eeuwijk et al., 2016; Malosetti et al., 2013, 2016; Bustos-Korts et al., 2009). This is also demonstrated in studies by Cooper et al. (2020); Mark et al. (2021) and Cooper et al. (2022) that focus on implementing management strategies to maximize crop productivity through combinations of Genotype–Management (G–M) technologies.

By testing new genotypes across multiple environments, breeders can gain valuable insights into how a variety's traits manifest and interact with the local environmental context. This information is essential for selecting cultivars that exhibit both high productivity and reliability, making them suitable for deployment across a wide geographical area (Smith et al., 2001b, 2019, 2021).

The conventional approach for analyzing MET data has relied on ANOVA-based methods, such as AMMI (additive main effects and multiplicative interaction) and GGE (genotype and genotype-by-environment interaction) analysis (Beeck et al., 2010; Zhang et al., 2020). These techniques have enabled researchers to obtain ANOVA results and gain insights into interactions. Additionally, they have facilitated data visualization through the use of biplot techniques.

However, these methods exhibit several inherent limitations that have become increasingly apparent as the complexity of MET experiments has grown. One key issue is that ANOVA-based methods may struggle to handle unbalanced and incomplete data structures, where some observations are missing or not all genotypes are present in each environment (Piepho, 1997; Smith et al., 2001a; Kelly et al., 2007). This is a common challenge in MET studies. Additionally, these methods may fail to adequately account for non-genetic sources of variance associated withenvironmental factors or experimental design effects, which can significantly influence the observed phenotypic performance (Smith et al., 2005). Furthermore, the ANOVA-based analysis commonly follows a two-stage modeling approach. This involves first analyzing the data within each environment, followed by a second stage of combining the results across environments for the G × E interaction analysis (Piepho et al., 2012; Smith et al., 2005). This approach can lead to a loss of information and less effective extraction of insights from the complex MET datasets.

Linear mixed model-based approaches have emerged as a more efficient methodology for the analysis of MET data. Recently, these mixed model approaches have become predominant, as they provide a flexible framework that can easily handle incomplete data and appropriately model the non-genetic variances between and within environments. This includes accounting for spatial variation within environments and error variance heterogeneity across environments (Smith et al., 2001a). The error variation within environments can be modeled using the approach of Gilmour et al. (1997), which appropriately models the three patterns of spatial trends associated with the field of trials: local, extraneous, and global trends.

Smith et al. (2001a, 2005) extended the G × E analysis by employing Factor Analytic Multiplicative Mixed (FAMM) models. These models assume random genotype effects and fixed environment effects, and use a one-stage analysis approach, where the models for residual effects are estimated simultaneously with the models for G × E effects. A key feature of the Factor Analytic (FA) model for MET data is its importance for the estimation of the associated variance structure for G × E effects. The FA model provides a good and parsimonious approximation to the unstructured form, and is generally more computationally robust (Kelly et al., 2007). Additionally, the best linear unbiased predictions (BLUPs) of the G × E effects and estimations of loadings and scores can be obtained, enabling bi-plot analysis to better understand the G × E interaction patterns.

The objective of this research is to explore the potential of linear mixed model-based approaches, particularly FAMM models, as a high-performing alternative to the traditional randomized complete block (RCB) designs analysis for effectively analyzing MET data and extracting meaningful insights from the complex G × E interactions. By leveraging linear mixed model-based methodology, this research aims to demonstrate how the non-genetic variances within and between environments can be appropriately modeled, including the use of the Gilmour et al. (1997) approach for capturing spatial trends and error variation. Furthermore, the application of FAMM models is explored, as these approaches provide advantages in estimating the variance structure of G × E effects and enabling more informative visualizations. The ultimate goal is to provide researchers and breeders with a robust and efficient analytical tool for extracting meaningful insights from MET data, ultimately supporting the development of high-performingand adaptable crop varieties.

## 2 Data and methods

### 2.1 Motivating data

The study utilized 10 MET datasets from the national variety trial (NVT) series conducted by the Ethiopian Institute of Agricultural Research (EIAR). All trials were laid out in RCB design with a rectangular array of plots, and were carried out across various locations in Ethiopia between 2014 and 2020. The trials were conducted by the research programs for common bean, chickpea, wheat, and maize. Each trial had a minimum of two replicates per entry. The terms “trial” and “environment” are used interchangeably, referring to a unique year-location combination. The trait analyzed in this study was harvested grain yield, measured in tons per hectare. There was a high degree of concurrence, or overlap, between entries both within and across years. The name of each dataset was designated using the first letter of the trial series name, two letters for the crop name, and the last two digits of years for the duration of the trial series (Table 1). Both complete datasets, where all entries were grown in all trials, and incomplete datasets were considered in the analysis. The complete datasets included LCB14-16, ACB14-16, SCB14-16, and BWT19-20. The total number of environments (year-location combinations) ranged from 8 to 16 across the different datasets.

**Table 1:** Summary of ten MET datasets.

**S.N**	**Programs**	**Dataset**	**Env**	**Entry**	**Entry.min**	**List of trials' dimension (Column × Row)**
1	Common bean	LCB14-16	9	16	16	(4 × 12)
2	Common bean	ACB14-16	12	16	16	(4 × 12)
3	Common bean	SCB14-16	9	16	16	(4 × 12)
4	Common bean	LCB19-20	8	117	28	(15 × 6), (15 × 9), (15 × 15), (15 × 22)
5	Common bean	SCB19-20	10	101	30	(15 × 6), (15 × 8), (18 × 11), (15 × 20)
6	Common bean	BCB15-18	15	18	16	(4 × 12), (6 × 9)
7	Chickpea	DCP14-16	16	27	12	(5 × 15), (14 × 4)
8	Chickpea	DCP16-18	14	27	12	(14 × 4), (15 × 5)
9	Maize	IHMZ20	8	32	26	(6 × 16)
10	Wheat	BWT19-20	9	50	50	(10 × 16)

Env, total number of environments; Entry, total number of entries; Entry.min, minimun number of entries grown in all trials.

### 2.2 Statistical models

ANOVA-based models have long been used for the analysis of MET data, providing a foundational approach to understanding genotype performance across different environments. The general statistical models for these techniques allow for the estimation of mean effects, genotype interactions, and random error components. Recently, linear mixed model-based approaches have become increasingly useful for analyzing complex MET data sets, as they can incorporate both fixed and random effects, accommodating variability in the data. In this section, we first present the general statistical model for ANOVA-based methods, followed by the statistical models for linear mixed model-based approaches, emphasizing their statistical applications in MET data analysis.

#### 2.2.1 ANOVA based models

The base-line statistical model for MET data analysis can be written as


(1) $$\begin{array}{c} \begin{array}{c} y_{ikj}=\eta_{ij}+\beta_{kj}+\varepsilon_{ikj}\\ \eta_{ij}=\mu +\alpha_{i}+\delta_{j}+\gamma_{ij} \end{array} \end{array}$$

where *y*_*ijk*_ is yield of the *i*^th^ entry of replicate block *k* in environment *j* (*i*=1, 2…*m, j*=1,2…*t, k*=1,2…r), η_*ij*_ is the empirical/least-square mean effect of entry *i* in environment *j*, μ is an overall mean effect, α_*i*_ is the main effect for genotype *i*, β_*kj*_ is the block effect at trial *j*, γ_*ij*_ is the interaction effect for genotype *i* in trial *j*, ε_*ikj*_ is the random error effect for genotype *i* in replicate block *k* of trial *j*, assumed to be N(0,σ2). The analysis of this model follow the approaches of two stage data analysis, in which the two-way table means η_*ij*_ are estimated first from the individual trial's analysis, and then the G × E analysis using GGE or AMMI model. The models for the second stage analysis can be written as


(2) $$\begin{array}{c} \gamma_{\text{ij}}=\left(\eta_{ij}-\left(\mu +\alpha_{i}+\delta_{j}\right)\right)=\overset{c}{\underset{i=1}{\sum }}\lambda_{l}\tau_{il}\theta_{jl}+\zeta_{ij} \end{array}$$

(3) $$\begin{array}{c} \left(\alpha_{l}+\gamma_{ij}\right)=\left(\eta_{ij}-\left(\mu +\delta_{j}\right)\right)=\overset{c}{\underset{i=1}{\sum }}\lambda_{l}\tau_{il}\theta_{jl}+\zeta_{ij} \end{array}$$

where *l* = 1, 2, . . ., *c*, λ_*l*_ is the singular value of the *l*^*th*^ multiplicative or principal component (PC), with *c* ≤ min(m−1, t), τ_*il*_ is the eigenvector of genotype i for PC *l*, θ_*jl*_ is the eigenvector of environment *j* for PC *l*, and ζ_*ij*_ is the residual associated with genotype *i* in environment *j*, assumed to be NID(0, σ^2^/r) where r is the number of replications within an environment. The models are subject to the constraints λ1 ≥ λ2, ..., λ*c* ≥ 0 and orthogonally constraints on the τ_*il*_ scores, that is $\overset{c}{\underset{i=1}{\sum }}\lambda_{l}\tau_{il}\tau_{i^{\prime }l}$ = 1 if *i* = *i*′ and $\overset{c}{\underset{i=1}{\sum }}\lambda_{l}\tau_{il}\tau_{i^{\prime }l}$ = 0 if *i* ≠ *i*′ with similar constraints on the θ_*jl*_ scores by replacing symbols (*i*, m, τ ) with (*j, s*, θ). AMMI analysis may use the model in Equation 2 whereas GGE analysis may use the model in Equation 3.

#### 2.2.2 Linear mixed models

A general form of linear mixed model for the *n* × 1 vector y of individual plot yields combined across trials can be written as


(4) $$\begin{array}{c} \text{y}=X\tau +Z_{g}u_{g}+Z_{o}u_{o}+\varepsilon \end{array}$$

where τ is the *a* × *1* vector of fixed effects, *u*_*g*_ is an *mt* × *1* vector of random *G* × *E* effects with associated design matrix *Z*_*g*_, *u*_*o*_ is a *b x 1* vector of (non-genetic) random effect with corresponding design matrix *Z*_*o*_, ε is the *n* × *1* vector of residual error across all trials. Some statistical assumptions are made about the random terms of the general linear mixed models. Thus, we assume that *u*_*g*_, *u*_*e*_ and ε are mutually independent and have a multivariate normal distribution with zero means vectors and variance matrices *var(u*_*g*_*)* = *Gg, var(u*_*e*_*)* = *Go* and *var*(ε) = R.

The random non-genetic effects *u*_*o*_ can be considered as sub-vectors ${u_{oj}}^{\left(b_{j}\times 1\right)}$ for each trial, where *b*_*j*_ is the number of random terms for trial *j*. These random terms are based on the terms for the blocking structure (e.g., replicate blocks or rows and columns of the field). In the analysis of MET data, the sub-vectors of *u*_*o*_ are typically assumed to be mutually independent, with variance matrix *G*_*oj*_for trial j that has a block diagonal form. Thus, there is a variance matrix$G_{o}={{⊕}^{t}}_{j}G_{oj}$ for the set of none-genetic effects at each trial *j*.

The variance matrix for resdual effects is assumed to be $R={⊗}_{j=1}^{t}R_{j}$ where *R*_*j*_ is the variance matrix for the *j*^th^ trial. Individual trial residual effects can be analyzed employing spatial methods of analysis that account for local or plot-to-plot variation. Each *R*_*j*_in this case will have its own spatial covariance structure (Gilmour et al., 1997). Varietal trials that have row by column arrangement and ordered as rows within columns allow separable spatial models of the form.


(5) $$\begin{array}{c} R_{j}=\sigma_{j}^{2}\Sigma_{c_{j}}\left(\rho_{c_{j}}\right)⊗\Sigma_{r_{j}}\left(\rho_{r_{j}}\right) \end{array}$$

where and Σ_*r*_*j*__ represent spatial correlation structures with parameters in ρ_*c*_*j*__ and ρ_*r*_*j*__ for the column and row directions, respectively. In both the column and row directions, we typically use an autoregressive spatial structure of order one, with ρ_*c*_*j*__ and ρ_*r*_*j*__ each containing a single autocorrelation parameter. For spatial auto-correlation in the row direction only, the model simplifies to $R_{j}=\sigma_{j}^{2}I_{n_{cj}}⊗\Sigma_{r_{j}}\left(\rho_{r_{j}}\right)$ where is the number of columns for trial j. Similarly, $R_{j}=\sigma_{j}^{2}\Sigma_{c_{j}}\left(\rho_{c_{j}}\right)⊗I_{n_{rj}}$ would be the reduced form for spatial auto-correlation in the column direction only where *n*_*rj*_ is the number of rows for trial j. We can have also no spatial covariance in either direction. Thus, the model simplified to an IID variance structure of the form.

Smith et al. (2001a, 2005) presented an alternative parsimonious model for *u*_*g*_using a factor analysis model to provide a variance structure for the genetic variance matrix. This model can adequately represent the nature of heterogeneous variances and covariances found to occur in most MET data. Thus, the *u*_*g*_ can be modeled with multiplicative terms. That is


(6) $$\begin{array}{c} \begin{array}{c} u_{g}=\left(\lambda_{1}⊗I_{m}\right)f_{1}+...+\left(\lambda_{d}⊗I_{m}\right)f_{d}+\xi \\ =\left(\Lambda ⊗I_{m}\right)f+\xi \end{array} \end{array}$$

where λ_*h*_ is the *t*×1 vector of loadings, *f*_*h*_ is the *m*×1 vector of factor scores (*h* = 1...*d*), ξ is the *mt*×1 vector of residuals, Λ is the *t*×*d* matrix of loadings {λ_1_ . . . λ_*d*_} and *f* is the *md*×1 vector of factor scores ${\left({f_{1}}^{\prime }{f_{2}}^{\prime }...{f_{d}}^{\prime }\right)}^{\prime }$. The random effects *f* and ξ are assumed to follow a normal distribution with zero mean vector and variance-covariance matrix.


(7) $$\begin{array}{c} \left[\begin{array}{cc} G_{f}⊗I_{m} & 0\\ 0 & \text{Ψ}⊗I_{m} \end{array}\right] \end{array}$$

where Ψ is a diagonal matrix of specific variances represents the residual variance not explained by the factor model, that is Ψ = *diag* (Ψ_1_ . . . Ψ_*t*_). The factor scores are commonly assumed to be independent and scaled to have unit variance, so that *G*_*f*_ = *I*_*d*_. The genetic effects *u*_*g*_ can be considered as a two dimensional (genotype by environment) array of random effects, and can be assumed to have a separable variance structure for the (*mt* × *mt* ) variance matrix *G*_*g*_ which can be written as


(8) $$\begin{array}{c} G_{g}={\text{G}}_{\text{e}}⊗G_{g} \end{array}$$

where *G*_*e*_ is the *t*×*t* genetic variance matrix representing the variances at each trial and covariances between trials, and *G*_*g*_ is the*m*×*m* symmetric positive definite matrix represents variances of environment effects at each genotype and the covariances of environment effects between genotypes. It is typically assumed that the varieties are independent and that *G*_*g*_ = *I*_*m*_. However, if the pedigree information of the varieties is available, other forms of *G*_*g*_ can be applicable (Smith et al., 2001a; Oakey et al., 2006, 2007). Based on Equation 2 the variance of genetic effects would be


(9) $$\begin{array}{c} \begin{array}{c} \mathrm{var}\left(u_{g}\right)=\left(\Lambda \Lambda^{\prime }+\text{Ψ}\right)⊗I_{m}\\ ={\text{G}}_{\text{e}}⊗I_{m} \end{array} \end{array}$$

Thus, the FA model approach results in the following form for *G*_*e*_.


(10) $$\begin{array}{c} G_{e}=\Lambda \Lambda^{\prime }+\text{Ψ} \end{array}$$

In the model, the variance parametric in these variance matrices are directly estimated using REML estimation method.

#### 2.2.3 Heritability formula

According to the methodology outlined by Cullis et al. (2006), the heritability ($H_{j}^{2}$) value for the *j*^th^ trial can be calculated from a generalized formula that is employed within the context of linear mixed model analysis. This formula is as follows:


(11) $$\begin{array}{c} H_{j}^{2}=1-\frac{A_{j}}{2{\sigma_{gj}}^{2}} \end{array}$$

where *A*_*j*_ is the average pairwise prediction error variance of genetic effects for the *j*^*th*^ environment and ${\sigma_{gj}}^{2}$ is the genetic variance at environment *j*.

### 2.3 Statistical inferences, analysis procedures and software

Fitting a linear mixed model involves estimating the values of the fixed effects (τ), a random G × E effects (*u*_*g*_), the random non-genetic effects (*u*_*o*_), as well as the variance-covariance parameters in G_g_, G_o_, and R. This estimation process comprises two interconnected steps. First, the variance parameters of the model are estimated using Residual Maximum Likelihood (REML), an approach introduced by Patterson and Thompson (1971). Second, the fixed and random effects are estimated using distinct techniques—Best Linear Unbiased Estimation (BLUE) is employed for the fixed effects, while Best Linear Unbiased Prediction (BLUP) is used for the random effects.

To assess the statistical significance of the random effects in the linear mixed model, the Residual Maximum Likelihood Ratio Test (REMLRT) can be utilized. However, it is important to note that the REMLRT is only applicable when comparing the fit of two nested models that share the same fixed effects structure. On the other hand, the significance of the fixed effects can be determined using the Wald test. The classic Wald statistic follows an asymptotic chi-squared distribution. Yet, this test has been found to be somewhat anti-conservative in certain scenarios, as reported by Butler et al. (2009). To address this issue, Kenward and Roger (1997) proposed an adjusted Wald statistic and an approximation based on the F-distribution, which have demonstrated improved performance across various settings.

The data analysis process began with the fitting of a randomized complete block (RCB) model. This initial model included random effects for block/replication and variety, and the residual correlation structure was specified as id(Column).id(Row), where 'id' refers to the identity matrix. The next step was to conduct a spatial analysis. First, a spatial model was fitted to the residuals from the RCB model. This spatial model used a separable autoregressive process in the column and row dimensions to account for local variation in the data.

After fitting the spatial model for local variation, a second spatial model was then fitted to capture the extraneous variation along the column and row dimensions. In this model, only the significant terms for local variation were retained. To assess the statistical significance of the fitted spatial models, both for the local and extraneous variation, the Residual Maximum Likelihood Ratio (REMLR) test was employed. Finally, spatial models were fitted to account for the global variation in the data, and the significance of these global spatial models was evaluated using the Wald test.

The other analysis procedure was a spatial + G × E analysis, which was done by incorporating the spatial terms identified in the spatial analysis and modeling the G × E effects using model fitting procedures demonstrated by De Faveri (2013) and Smith et al. (2015). In this analysis, a combined model was first fitted, which is a combined form of individual trial models constructed in the spatial analysis. This combined model forms the basis of a sequence of models to be fitted for the G × E analysis, and it helps to organize the trial-specific models in a combined form and to confirm the presence of genetic variance in each trial. If any trial is found to have no genetic variance, it would be excluded from the multi-environment trial (MET) data analysis.

Factor Analytic (FA) models were then considered, while maintaining the spatial models as specified in the combined model. The adequacy of the FA models of several factors (h) was formally tested, as they are fitted within a mixed model framework. A model with h factors, denoted as FA-h, is nested within a model with h+ 1 factors. The models were compared, such as FA-1 vs. FA-2, FA-2 vs. FA-3, and so on. Both the Residual Maximum Likelihood Ratio Test (REMLRT) and total percentage of the G × E variance (%var) explained by factor components were used to identify the final plausible FA models.

The licensed version of the ASReml-R statistical software package was used to fit all models analyzed in this study (Butler et al., 2009). ASReml-R is a specialized software application designed for fitting linear mixed models, which was well-suited for the data and research questions addressed here.

## 3 Results

### 3.1 Spatial analysis

Table 2 presents the results of the Wald test, which was used to assess global spatial variation, as well as the REMLR test, which was employed to detect local and extraneous spatial variation. The analysis revealed several instances of significant spatial variations across the trials. For the trial AN19CBN2, there was notable local spatial variation detected along both the row and column dimensions (*p* < 0.001). Local spatial variation was also present for the trial 2015CNVT-D-CD, which exhibited significant variation along the column dimension (*p* = 0.022), as well as for the trial AA20BWNE, which showed local spatial variation along the row dimension (*p* = 0.001).

**Table 2:** Summary of spatial analysis for four trials: spatial variation, fitted model term, Wald and REML test statistic and *P*-value.

**Dataset**	**Trial**	**Spatial variation**	**Model terms**	**Wald^a^/REMLR^b^ test statistics**	***P*-value**
SCB19-20	AN19CBN2	Local	ar1(Column):id(Row)	26.71	< 0.001
		Local	ar1(Column):ar1(Row)	15.57	< 0.001
DCP14-16	2015CNVT-D-CD	global	lrow	13.04	0.007
		Global	lcol	7.20	0.017
		Local	ar1(Column):id(Row)	5.25	0.022
IHMZ20	2020MZNVT- AS	Global	lcol	11.21	0.030
		Extraneous	Column	7.92	0.005
		Extraneous	Column +Row	5.14	0.023
BWT19-20	AA20BWNE	Extraneous	Row	13.98	< 0.001
		Extraneous	id(Column):ar1(Row)	18.28	< 0.001
ρ_cr_ = (0.28, 0.31); ρ_c_ = 0.56; ρ_r_ = 0.50				

^a^Test for global trend after significant terms for extraneous variation and local trend are fitted.

^b^Test for extraneous variation and local trend after significant terms for global trend are fitted ρ_cr_, ρ_c_, and ρ_r_ are estimates for autoregressive order 1(AR1) spatial correlation parameters at AN19CBN2 in the column and row direction, at 2015CNVT-D-CD in the column direction and at AA20BWNE in the row direction, respectively.

In terms of global linear spatial trends, these were found to be significant along both the row and column dimensions for the trial 2015CNVT-D-CD (*p* = 0.007 and *p* = 0.017, respectively) and along solely the row dimension for the environment 2020MZNVT-AS (*p* = 0.030). Extraneous spatial variation, which cannot be accounted for by linear trends, was detected for the environment 2020MZNVT-AS along both dimensions (*p* = 0.023) and for the trial AA20BWNE along the row dimension. The estimated AR1 correlations for the significant local spatial terms were all positive (ρ_cr_ = (0.28, 0.31); ρ_c_ = 0.56; ρ_r_ = 0.50).

**Figure 1:**
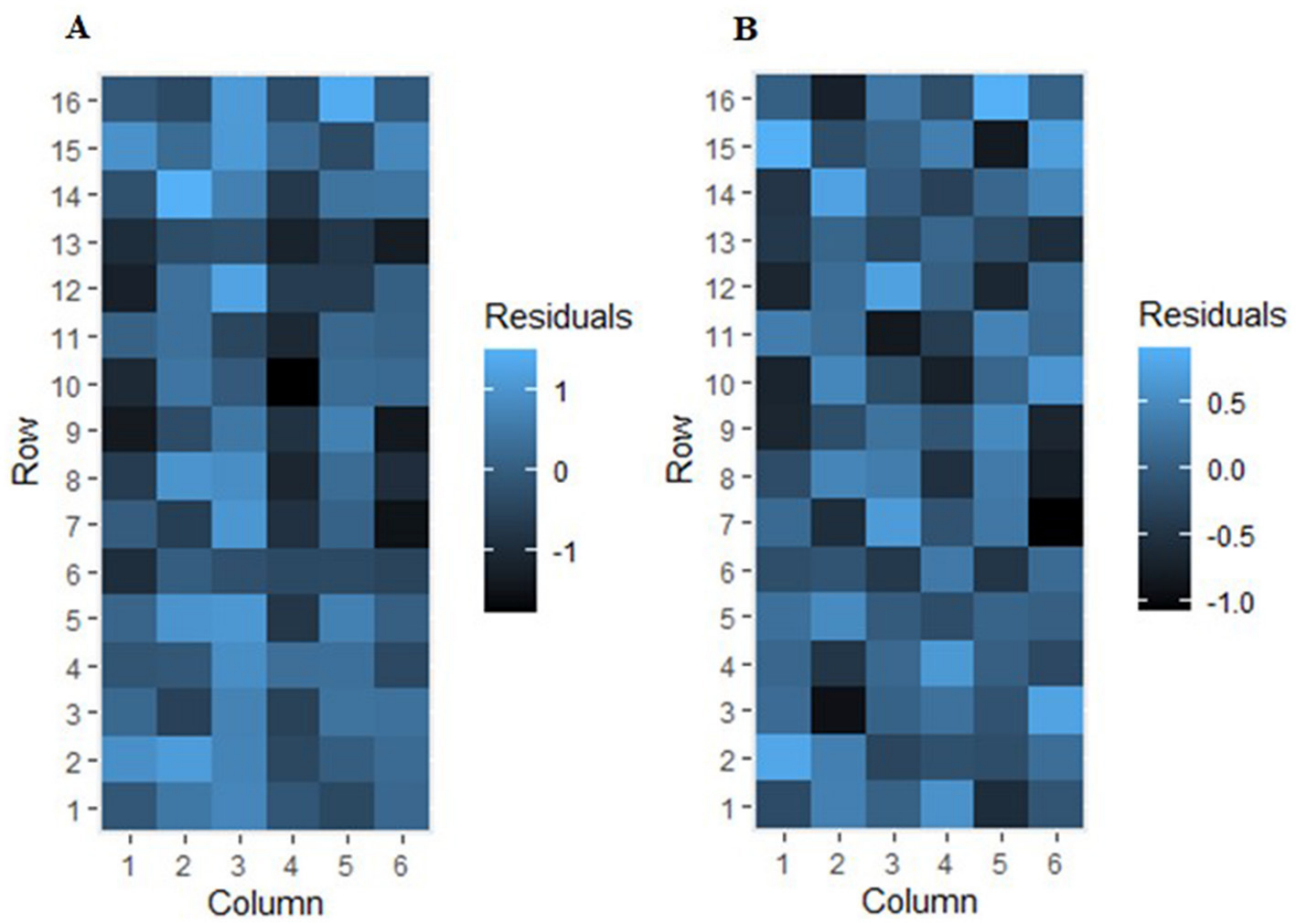
Residual variation heat map before **(A)** and after **(B)** spatial analysis for trial 2020MZNVT-AS.

The heat map visualization of the residual variation for the trial 2020MZNVT-AS, before and after the spatial analysis, is presented in Figure 1. This plot clearly reveals the presence of observable systematic spatial trends within the trial (Figure 1A). The observed spatial trends suggest the presence of extraneous variation that is not adequately accounted for in the initial model. Such systematic spatial patterns are indicative of the need to incorporate random row and column effects, as well as fixed linear global trends, into the linear mixed models (Figure 1B).

### 3.2 Spatial + G × E analysis

The results of the factor analytic (FA) model comparisons from the Spatial + G × E analysis for each dataset are presented in Table 3. This includes the residual log-likelihoods (LR), residual maximum likelihood ratio tests (REMLRT), and the total percentage of the G × E variance explained by the FA model (%var).

**Table 3:** FA model comparisons through the total percentage of the G×E variance (%var) explained by the FA components, residual log-likelihoods (LR), and residual maximum likelihood ratio tests (REMLRT).

**Dataset**	**FAs models**	**%var**	**LR**	**REMLRT**	**Final model**
LCB14-16	FA-1	27.6	253.1	-	FA-3
	FA-2	49.3	263.8	< 0.001	
	FA-3	80.0	267.8	0.091	
ACB14-16	FA-1	18.9	293.3	-	FA-5
	FA-2	33.0	299.2	0.026	
	FA-3	52.3	304.3	0.082	
	FA-4	59.9	308.7	0.058	
	FA-5	65.1	312.3	0.035	
	FA-6	88.9	315.5	0.342	
SCB14-16	FA-1	98.3	266.4	-	FA-2
	FA-2	100	271.2	< 0.001	
LCB19-20	FA-1	57.6	−16.2	-	FA-3
	FA-2	61.1	−5.4	< 0.001	
	FA-3	80.8	0.78	< 0.001	
	FA-4	94.5	1.74	0.281	
SCB19-20	FA-1	66.8	28.6	-	FA-3
	FA-2	73.4	37.8	< 0.001	
	FA-3	78.6	47.0	< 0.001	
	FA-4	84.0	49.4	0.199	
BCB15-18	FA-1	17.7	552.8	-	FA-6
	FA-2	28.6	563.9	< 0.001	
	FA-3	62.2	576.7	< 0.001	
	FA-4	68.5	588.9	0.004	
	FA-5	82.2	597.1	0.009	
	FA-6	93.4	601.6	0.037	
	FA-7	93.4	602.8	0.757	
DCP14-16	FA-1	54.7	178.8	-	FA-3
	FA-2	73.6	195.2	< 0.001	
	FA-3	87.6	206.1	0.001	
	FA-4	91.3	211.7	0.072	
DCP16-18	FA-1	65.5.	208.9	-	FA-4
	FA-2	80.6	220.2	0.001	
	FA-3	84.3	226.3	0.023	
	FA-4	92.2	232	0.040	
	FA-5	98.2	234.2	0.450	
IHMZ20	FA-1	56.9	−606.1	-	FA-3
	FA-2	72.2	−599.9	0.014	
	FA-3	88.2	−594.7	0.008	
	FA-4	93.6	−593.6	0.454	
BWT19-20	FA-1	48.2	−206.4	-	FA-4
	FA-2	62.0	−186.2	< 0.001	
	FA-3	64.9	−178.7	0.007	
	FA-4	69.1	−174.9	0.046	
	FA-5	81.3	−174.1	0.531	

The analysis revealed that increasing the order of the FA model consistently improved the total percentage of G × E variance explained across all datasets. As the order of the FA model was increased, the significance level of the REMLRT generally decreased across most datasets. However, this pattern did not hold true for the ACB14-16 dataset, where the lower-order FA-2 and higher-order FA-5 models were found to be statistically significant, while the intermediate FA-3 and FA-4 models did not reach significance levels.

Notably, the total percentage of G × E variance explained by the last significant order of the FA model was generally >65% for each dataset, with the exception of LCB14-16. For the LCB14-16 data set, the second-order FA-2 model accounted for 49.3% of the G × E variance, which was still statistically significant. In contrast, the higher-order FA-3 model showed a substantial improvement, explaining 80.0% of the G × E variance, but this was not statistically significant.

In the case of the SCB14-16 data set, the analysis determined that the FA-2 model accounted for a remarkable 100% of the G × E variance, with a highly significant *p* < 0.001. The third-order FA-3 model was also found to be a plausible model for the G × E analysis in the datasets LCB19-20, SCB19-20, DCP14-16, and IHMZ20, as it explained at least 65% of the G × E variance with *p* ≤ 0.081. For the DCP16-18 and BWT19-20 data sets, the fourth-order FA-4 model was selected as the final model, accounting for 92.2 and 69.1% of the G × E variance, respectively, with statistically significant *p*-values. In the case of the ACB14-16 data set, the FA-5 model was chosen as the final model, explaining 65.1% of the G × E variance with a p-value of 0.035. Finally, for the BCB15-18 data set, the higher-order FA-6 model was determined to be the most appropriate, accounting for 93.4% of the G × E variance with a p-value of 0.037.

The average genetic and error variance estimates for each dataset, derived from the RCB, spatial, and spatial + G × E analysis, are presented in Table 4. In the RCB analysis, the average genetic variance estimates ranged from 0.050 to 0.984, and the average error variance estimates ranged from 0.072 to 1.746. The spatial analysis produced average genetic variance estimates ranging from 0.049 to 1.009, and average error variance estimates with a minimum of 0.065 and a maximum of 1.571. From the spatial + G × E analysis, the average genetic variance estimates had a minimum of 0.052 and a maximum of 1.057, while the error variance estimates fell within the range of 0.061 to 1.568. Across all three methods of analysis, the 9IHMZ20 data set exhibited the highest average genetic and error variance, while the 6BCB15-18 data set had the lowest average genetic and error variance.

**Table 4:** A summaries of results from the RCB, Spatial, Spatial + G × E analysis for each MET dataset.

	**Genetic variance**			**Error variance**		
**Dataset**	**RCB**	**Spatial**	**Spatial** + **G** × **E**	**RCB**	**Spatial**	**Spatial** + **G** × **E**
1LCB14-16	0.067	0.071	0.075	0.106	0.087	0.087
2ACB14-16	0.120	0.120	0.125	0.144	0.140	0.134
3SCB14-16	0.167	0.159	0.168	0.134	0.135	0.126
4LCB19-20	0.211	0.261	0.256	0.718	0.474	0.468
5SCB19-20	0.177	0.188	0.237	0.320	0.310	0.310
6BCB15-18	0.050	0.049	0.052	0.072	0.065	0.061
7DCP14-16	0.089	0.107	0.122	0.277	0.204	0.194
8DCP16-18	0.087	0.116	0.126	0.273	0.238	0.230
9IHMZ20	0.984	1.009	1.057	1.746	1.571	1.568
10BWT19-20	0.705	0.762	0.763	0.385	0.317	0.317

The estimates of genetic variance were observed to improve with the application of spatial analysis, as compared to RCB analysis. More importantly, the spatial + G × E analysis led to substantial improvements in the genetic variance estimates for most of the datasets examined. The spatial analysis provided notably smaller estimates of the error variance compared to the RCB analysis. Interestingly, the spatial + G × E analysis was able to significantly reduce the residual variability, outperforming both the RCB and the spatial methods analysis.

**Figure 2:**
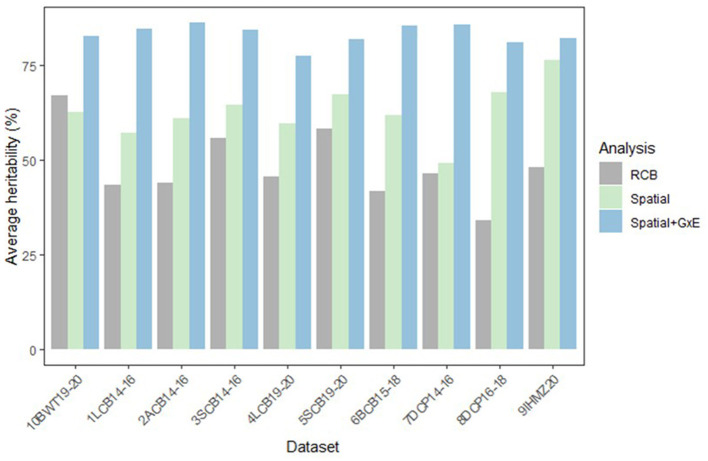
Average heritability of yield for each dataset using the mixed model analysis: RCB, spatial, and Spatial+G×E analysis.

Figure 2 presents the average heritability estimates of yield for each dataset, derived from RCB, Spatial, and Spatial + G × E analysis. The Spatial analysis consistently provided higher heritability estimates compared to the RCB analysis across all the datasets. Furthermore, the spatial + G × E analysis further highly improves the heritability estimates compared to the RCB and Spatial analysis across all the datasets. The extent of improvement from the spatial and spatial + G × E analysis varied among the datasets. Larger improvements were observed for datasets with relatively higher data volumes, such as DCP14-16 (16 trials, 27 entries) and LCB19-20 (8 trials, 117 entries), compared to datasets with lower data volumes, such as LCB14-16 (8 trials, 16 entries) and IHMZ20 (8 trials, 32 entries). While increasing the volume of data generally improves the reliability of statistical estimates and minimizes the risk of misleading conclusions from small sample sizes, it is important to recognize that more data does not inherently ensure that advanced data analysis methods will outperform conventional approaches. The effectiveness of advanced techniques, such as Spatial + G × E analysis, largely depends on the presence of significant factors or sources of variability that need modeling, such as spatial variability or strong G × E interactions.

In our study, we observed that the spatial + G × E method significantly enhanced the analysis, with improvements particularly evident in larger datasets. This finding aligns with the understanding that spatial variability and strong G × E interactions are often more pronounced in larger datasets, a trend frequently observed in Ethiopia's crop MET data, where notable G × E interactions and plot variability are common, as highlighted by Woldemeskel and Fenta (2022) and Taye (2005). Thus, while accumulating more data is beneficial, the true advantage lies in the capacity of advanced methods to capture and model specific sources of variation, especially in larger datasets where these factors become more prominent.

**Figure 3:**
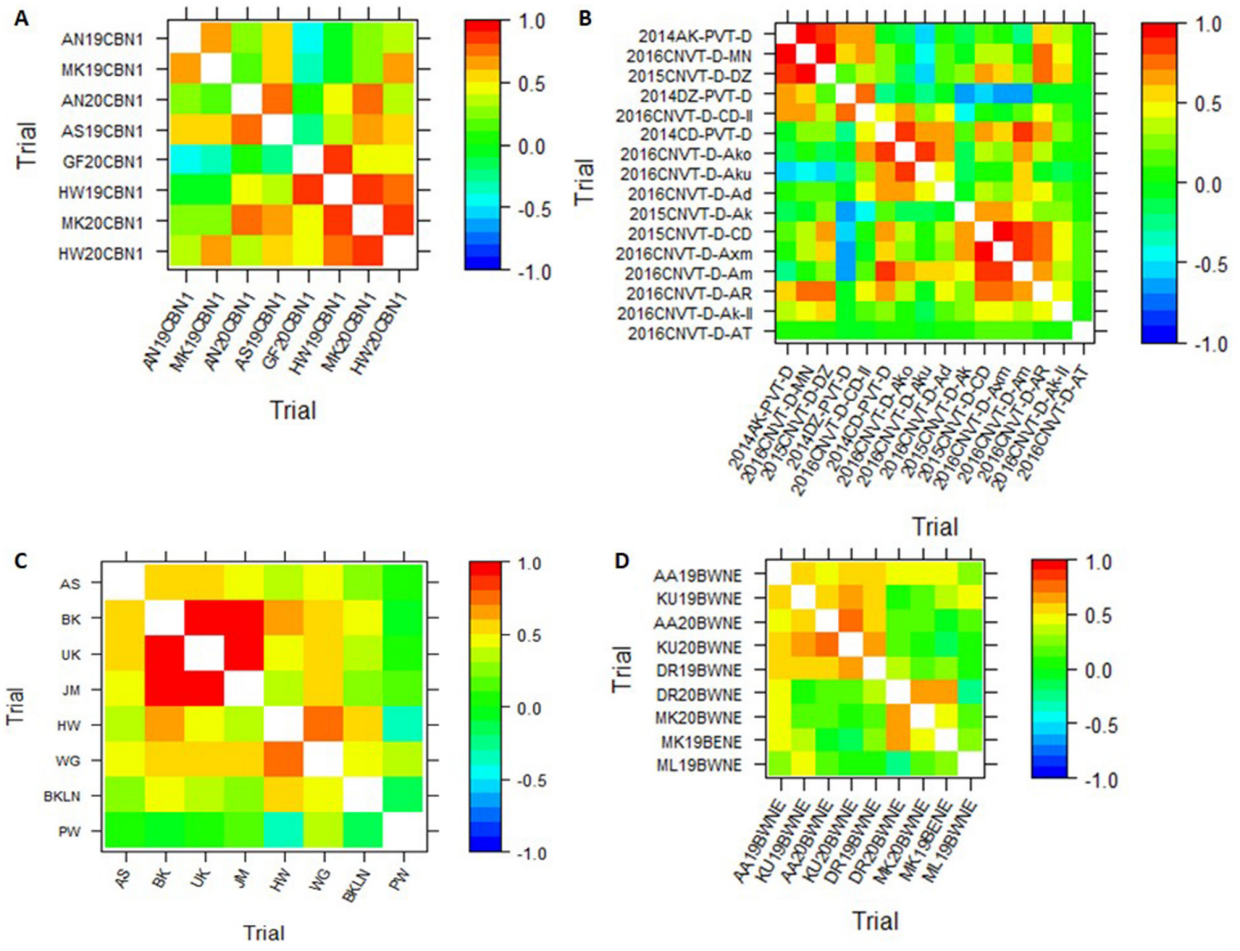
Heat map representation of the genetic correlation matrix from spatial+G×E analysis for the datasets SCB19-20 **(A)**, DCP14-16 **(B)**, IHMZ20 **(C)**, and BWT19-20 **(D)**.

**Figure 4:**
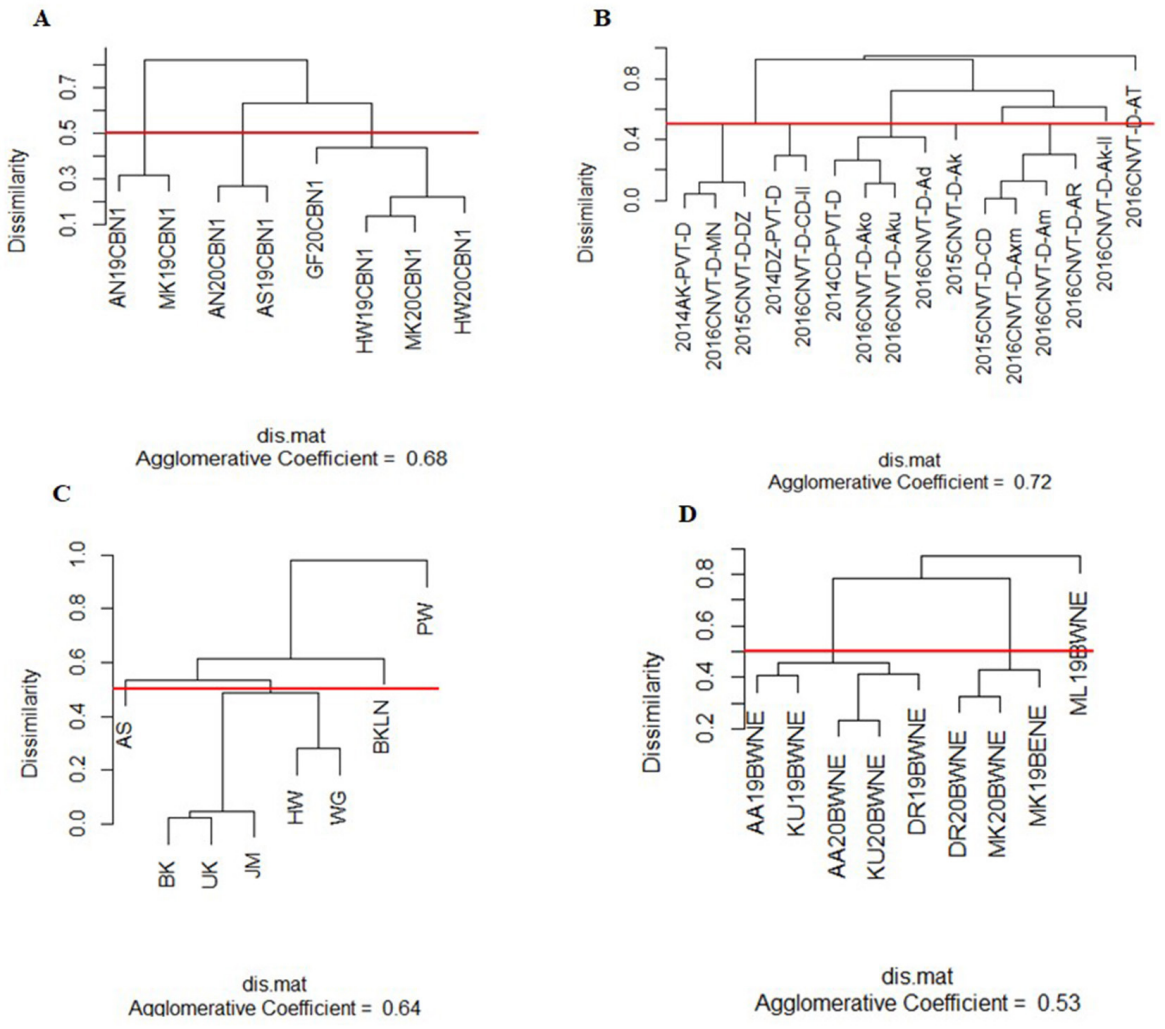
Dendrogram representation of the dissimilarity matrix from spatial+G×E analysis for the datasets SCB19-20 **(A)**, DCP14-16 **(B)**, IHMZ20 **(C)**, and BWT19-20 **(D)**.

The visualization techniques from the spatial + G × E analysis are presented in Figures 3, 4. Figure 3 displays the heat map representation of the genetic correlation matrix for the dataset SCB19-20 (A), for dataset DCP14-16 (B), for the dataset IHMZ20 (C), and for the dataset BWT19-20 (D). Figure 4 presents the dendrogram representation of the dissimilarity matrix for those same datasets.

The genetic correlation heat map (Figure 3) shows strong positive correlations among some trials within each dataset, denoted by the deep red coloration. Conversely, weak positive and negative correlations are observed for other trial pairings, as indicated by the yellow hues. Additionally, the heat map reveals strong negative correlations between certain trials, represented by the deep blue shading. This is particularly evident in the DCP14-16 dataset (Figure 4B). Turning to the dendrogram visualization (Figure 4), the dissimilarity cut-off at 0.5 delineates three distinct trial clusters within the SCB19-20 dataset (Figure 4A) and BWT19-20 datasets (Figure 4D), four clusters in IHMZ20 (Figure 4C), and five clusters in DCP14-16 (Figure 4B).

## 4 Discussion

The spatial analysis the individual trial data revealed several key insights. The presence of significant local, extraneous and global spatial variation, as detected by the Wald and REMLR tests, highlights the critical importance of accounting for these spatial effects when analyzing data from multi-environment trials. The positive estimated AR1 correlations, indicating that nearby plots tend to be more similar than those located further apart, underscores the need to incorporate appropriate spatial covariance structures in the linear mixed models used for these analyses (Cullis et al., 1998; Stefanova et al., 2009).

The systematic spatial trends observed in the residual plots (Figure 1A) for the 2020MZNVT-AS trial clearly demonstrate the value of including random row and column effects, as well as fixed linear global trends, in the modeling approach. This aligns with the recommendations from previous studies on the analysis of multi-environment trials, which have emphasized the importance of capturing both local and global spatial patterns to improve the accuracy and reliability of genotypic evaluations (Möhring and Piepho, 2009; Piepho et al., 2012; Smith et al., 2005).

The findings from the current study are consistent with the work of Qiao et al. (2000), who highlighted the importance of evaluating experimental designs and spatial analyses in wheat breeding trials. Similarly, the application of enhanced statistical models for the analysis of multi-environment trials in finger millet (Tesfaye et al., 2023) and common bean (Argaw et al., 2024) underscores the broader relevance and applicability of the spatial modeling approaches employed in this study across various crop varietal evaluation trials.

Furthermore, the use of spatial mixed models for varietal selection field trials, as discussed by Woldemeskel and Fenta (2022), reinforces the practical utility of the methods demonstrated here. The insights gained from the spatial analysis, such as the identification of significant local and global spatial variation, can directly inform the selection of high-performing genotypes and the optimization of experimental designs in future trials. This can lead to more accurate assessments of genotypic performance and ultimately contribute to the development of improved crop varieties (Kelly et al., 2007).

The results of the spatial + G × E analysis across the multiple datasets provide valuable insights into the underlying patterns of the G × E effects. One of the key findings is the consistent superiority of the factor analytic (FA) modeling approach over the conventional RCB analysis in capturing the G × E variance. As the order of the FA models increased, meaning more factors were incorporated, the total percentage of G × E variance explained also increased (Table 3). This observation aligns with the theoretical foundations and empirical evidence presented in previous studies (Smith et al., 2001a, 2005; Burgueño et al., 2008; Meyer, 2009). This supports the notion that higher-order FA models are better able to effectively represent the complex and multidimensional nature of G × E effects.

However, the optimal FA model order was found to be dataset-dependent, as demonstrated by the ACB14-16 dataset, where the intermediate FA-3 and FA-4 models did not reach statistical significance, while the lower-order FA-2 and higher-order FA-5 models were significant (Table 1). This observation suggests that the appropriate model complexity may vary based on the specific characteristics of the data, such as the magnitude and structure of the G × E effects. This scenario could be associated with the implications of trial locations. Even with the best analytical methods, poorly distributed trials can complicated the MET data analysis. This leads us to the concept of the target population of environments (TPE). The TPE framework emphasizes the need for trials to be representative of the environments where the genotypes will be deployed. Misalignment between trial locations and the intended target environments can result in inaccurate assessments of genotypic performance (Cooper et al., 2020, 2021; Hajjarpoor et al., 2022). However, the identification of the optimal FA model order is a crucial step, as emphasized by De Resende and Thompson (2004) and Smith et al. (2015) as it ensures the most parsimonious yet informative representation of the G × E structure.

Compared to the standard RCB analysis, the spatial analysis improved estimates of genetic variance, while the spatial + G × E analysis led to even greater improvements in genetic variance estimates for most datasets. This potential can be attributed to the spatial + G × E analysis's ability to extract the genetic effect that is confounding with the residual effect and the effects stored in the G × E interactions. Notably, the spatial analysis reduced estimates of error variance, and the spatial + G × E analysis (Table 4) was able to substantially minimize residual variability, outperforming both the RCB and spatial-only methods.

The integration of spatial variability into the G × E analysis through the spatial + G × Emodeling approach has led to substantial improvements in heritability estimates across all datasets compared to the RCB and spatial analyses (Figure 2). This supports the findings of Smith et al. (2015), who demonstrated the ability of factor analytic mixed models to enhance the estimation of genotypic effects and heritability. Interestingly, the extent of improvement was more pronounced in datasets with relatively larger data volumes, such as DCP14-16 and LCB19-20, compared to smaller datasets like LCB14-16 and IHMZ20. This observation highlights the importance of data quality and quantity in effectively capturing the underlying patterns of G × E interactions and larger datasets provide more information about the complex G × E structure, allowing the factor analytic models to better estimate the genotypic effects and associated heritability. The advancement lies in demonstrating the superiority of the factor analytic model across different sizes of MET datasets, particularly in its ability to effectively investigate G × E interactions. This approach allows for a comprehensive understanding of how varying dataset sizes influence the model's performance to uncover underlying patterns in G × E relationships.

The visual representations, including the genetic correlation heat map and dendrogram, offer intuitive and informative insights into the trial relationships within each dataset. The observed patterns of strong positive, negative, and weak correlations in the heat maps (Figure 3), as well as the distinct trial clusters identified in the dendrograms (Figure 4), reflect the complex structure of the G × E interactions, as discussed in previous studies (Cullis et al., 2010; Argaw et al., 2024). These visual aids can assist breeders and researchers in better understanding the spatial and genotypic patterns, which can inform decision-making processes related to cultivar selection, target environment identification, and trial design optimization.

## 5 Conclusion

Our research constitute a noteworthy contribution to the field by demonstrating the effectiveness of mixed model-based approaches, particularly spatial + G × E analysis, in enhancing MET analysis. Unlike previous studies, we provide comparative insights across various MET datasets, revealing that the optimal order of the FA model for G × E effects varies depending on the specific dataset. Furthermore, our application of advanced linear mixed model methods to Ethiopia's crop MET data—characterized by notable G × E interactions and plot variability—offers critical insights that are highly relevant to crop breeding programs in the region.

The spatial analysis of the individual trial data revealed significant local, extraneous, and global spatial variation, underscoring the need to use mixed model spatial analysis for multi-environment trial data analyses. The positive estimated AR1 correlations indicate that nearby plots tend to be more similar, further emphasizing the importance of capturing local spatial patterns to improve the accuracy and reliability of genotypic evaluations. Employing these spatial modeling techniques in future trials is recommended to account for the inherent spatial variability, which can enhance the genetic gain achieved through variety selection.

The results of the spatial + G × E analysis showcased the value of factor analytic (FA) modeling in effectively representing the complex and multidimensional nature of G × E effects. The consistent superiority of the FA approach over the traditional RCB analysis supports the use of FA models, though the optimal model order may vary across datasets. The approach is significant because it applies the FA model to MET datasets of varying sizes, offering insights into GxE effects, and improves our understanding of how dataset size impacts model performance, enabling more accurate identification of underlying patterns in G × E relationships.

Researchers are encouraged to explore the application of these spatial + G × E modeling techniques across a broader range of crop breeding evaluation programs to further validate their benefits in increasing the efficiency of variety selection and improving genetic gain.

The integration of spatial variability into the G × E analysis led to substantial improvements in heritability estimates across all datasets. This highlights the ability of factor analytic mixed models to enhance the estimation of G × E effects and heritability, with more pronounced improvements observed in larger datasets. By accurately partitioning the sources of variation, these advanced statistical models can lead to more reliable predictions of genotypic performance, ultimately contributing to greater genetic gain in crop improvement programs.

The insights gained from this study can contribute to the development of improved crop varieties and enhanced experimental designs in terms of establishing good trailing system that optimize statistical precision associated with the size future trials. Continued research and adoption of advanced spatial and G × E modeling techniques in crop breeding and evaluation programs will be crucial for driving progress in the creation of high-performing agricultural cultivars. By accounting for the complex spatial and G × E structures inherent in multi-environment trials, researchers and breeders can improve the estimation of genetic gain and make more informed decisions to deliver high-performing crop varieties to farmers and consumers.

## Data Availability

The raw data supporting the conclusions of this article will be made available by the authors, without undue reservation.
